# Assessment of breast cancer risk among Iraqi women in 2019

**DOI:** 10.1186/s12905-021-01557-1

**Published:** 2021-12-15

**Authors:** Hashim Talib Hashim, Mustafa Ahmed Ramadhan, Kabas Monther Theban, John Bchara, Ahed El-Abed-El-Rassoul, Jaffer Shah

**Affiliations:** 1 University of Baghdad, College of Medicine, Baghdad, Iraq; 2 Faculty of Medicine, Tishreen University, Latakia, Syria; 3 Faculty of Medical Sciences, Lebanese University, Hadath, Beirut, Lebanon; 4 College of Medicine, Drexel University, Philadelphia, USA

**Keywords:** Breast cancer, Gail Model, BCRAT, Assessment, 5 years' risk, Lifetime risk

## Abstract

**Background:**

Breast cancer is one of the most common cancers among women worldwide and the leading cause of death among Iraqi women. Breast cancer cases in Iraq were found to have increased from 26.6/100,000 in 2000 to 31.5/100,000 in 2009. The present study aims to assess the established risk factors of breast cancer among Iraqi women and to highlight strategies that can aid in reducing the incidence.

**Methods:**

1093 Iraqi females were enrolled in this cross-sectional study by purposive sampling methods. Data collection occurred from July 2019 to September 2019. 1500 women participated in the study, and 407 women were ultimately excluded. The questionnaire was conducted as a self-administrated form in an online survey. Ethical approval was obtained from the College of Medicine in the University of Baghdad. The Gail Model risk was calculated for each woman by the Breast Cancer Risk Assessment Tool (BCRAT), an interactive model developed by Mitchell Gail that was designed to estimate a woman’s absolute risk of developing breast cancer in the upcoming five years of her life and in her lifetime.

**Results:**

The ages of the participants ranged from 35 to 84 years old. The mean 5–year risk of breast cancer was found to be 1.3, with 75.3% of women at low risk and 24.7% of women at high risk. The mean lifetime risk of breast cancer was found to be 13.4, with 64.7% of women at low risk, 30.3% at moderate risk, and 5.0% at high risk. The results show that geographically Baghdad presented the highest 5-year risk, followed by Dhi Qar, Maysan, and Nineveh. However, the highest lifetime risk was found in Najaf, followed by Dhi Qar, Baghdad, and Nineveh, successively.

**Conclusion:**

Breast cancer is a wide-spreading problem in the world and particularly in Iraq, with Gail Model estimations of high risk in several governorates. Prevention programs need to be implemented and awareness campaigns organized in order to highlight the importance of early detection and treatment.

## Introduction

Breast cancer is one of the most common cancers among women worldwide, and the second leading cause of death in women after lung cancer [1]. Each year, nearly 2.09 million women are diagnosed with breast cancer and 627 000 die from the disease [2]. In Iraq, breast cancer is the most common cancer, and the leading cause of death among Iraqi women [3]. Breast cancer-related cases in Iraq were found to have increased from 26.6/100,000 in 2000 to 31.5/100,000 in 2009 [4]. Moreover, the age-related incidence rate in Iraq was found to be greater than that in Turkey, Iran, Saudi Arabia, and Bahrain, while less than that in Jordan and Kuwait [5].

Breast cancer risk factors are related to the female’s age, parity, family history of breast cancer, especially first-degree relatives, radiation exposure, smoking, and the genetic factors of BRCA1 and BRCA 2 gene mutations [6]. Awareness of the symptoms and early screening are important methods of reducing the risks associated with breast cancer. The American Cancer Society has developed guidelines for the prevention and early detection of breast cancer, where women with moderate-to-high risks of developing breast cancer are recommended to undergo regular screening mammography tests starting at the age of 45 years [7, 8].

There are many risk assessments models—such as the Gail Model, Claus Model, BRCAPRO Model, and Cuzick–Tyrer Model—that are used as tools to determine a female’s breast cancer-associated risk [9–12]. The Gail Model is the most widely used risk model, as it calculates both a 5-year risk and a lifetime risk of breast cancer. This risk is measured based on the woman’s age, age of menarche, age of first birth, family history, and number of biopsies conducted [11, 13]. The Gail Model has been validated in many countries and is used extensively in many studies as a tool to assess breast cancer-related risk [13].

This study aims to assess the established risk factors of breast cancer among Iraqi women and to highlight the benefits of certain strategies that can aid in controlling its incidence.

## Materials and methods

### The aim, design and setting of the study

Our study is a cross-sectional study, enrolling 1093 Iraqi females, out of an estimated population of 41 million, by purposive sampling methods from all of Iraq’s 18 governorates [14].

### The characteristics of participants or description of materials

The purpose of this study was explained to each participant before acquiring their consent to participate, and those who refused to participate were excluded. Women who had previously received chest radiation therapy for the treatment of Hodgkin Lymphoma were also excluded. Data collection occurred from July 2019 to September 2019, with 1500 women participating in the study from all of Iraq’s cities. 407 women were excluded for the following reasons: 150 refused to participate, 169 had already been diagnosed with breast cancer, and 88 had received chest radiation for the treatment of Hodgkin Lymphoma. As a result, only 1093 women were included the study.

The Gail Model was implemented to assess the risk of breast cancer among the study records. This model is known by the National Cancer Institute as BCRAT (Breast Cancer Risk Assessment Tool).

The questionnaire was conducted as a self-administrated form in an online survey, as well as face-to-face interviewing and a paper survey. A pilot study of 73 participants was first performed in order to test the validity and reliability of the Arabic version of the questionnaire. It was tested for validity by sending a translation of the questionnaire to six experts in the specialty—three from Baghdad Medical College, two from Dhi Qar Medical College, and one from Basra Medical College—that all accepted the format upon revision. The questionnaire was further tested for reliability by assessing the 5-year risk of the first 73 women upon administering the survey, and then repeating the survey with the same group at a later point of time to compare the predicted risk for each. The overall internal reliability (Cronbach’s α = 0.87) was high.

A structured questionnaire was used to collect sociodemographic data from participants, including number of children, occupation, educational level, monthly income, use of contraceptives, breast feeding, smoking, and physical activity. In addition, details were collected regarding risk factors for breast cancer, such as age, age at menarche, age at the 1^st^ live birth, number of previous breast biopsies, presence of atypical hyperplasia in any previous breast biopsy specimen, and history of breast cancer among the participant’s first-degree relatives (mother, daughter and sister). Participant race was also collected on the questionnaire, with three options relating to the three main races of Iraqi women (Arabic, Kurdish, and Turkmen).

Ethical approval was obtained from the College of Medicine in the University of Baghdad in order to conduct the study.

### The statistical analysis

A Student’s t-test was used to check for any significant differences between the mean values of two continuous variables. Multiple linear regression models were also used to estimate the effect of each variable on the 5-year and lifetime breast cancer risk. The level *P* < 0.05 was considered as the cutoff value for significance.

The Gail Model risk for each woman was calculated by Breast Cancer Risk Assessment Tool (BCRAT), an interactive model developed by Mitchell Gail for estimating a woman’s absolute risk of developing invasive breast cancer in both the upcoming five years of her life and her entire lifetime.

The Gail Model calculates the probability of a woman at age **α** who has age-related relative risk r(t). The woman may develop breast cancer by age **α + τ** according to the following equation:

$${\varvec{p}}\left\{{\varvec{a}}.{\varvec{\tau}}.{\varvec{r}}\left({\varvec{t}}\right)\right\}=\underset{{\varvec{a}}}{\overset{{\varvec{a}}+{\varvec{\tau}}}{\int }}{{\varvec{h}}}_{1}\left({\varvec{t}}\right){\varvec{r}}({\varvec{t}}){{\varvec{e}}}^{-\underset{{\varvec{a}}}{\overset{{\varvec{\tau}}}{\int }}{{\varvec{h}}}_{1\left({\varvec{u}}\right){\varvec{r}}\left({\varvec{u}}\right){\varvec{d}}{\varvec{u}}}}\left\{{{\varvec{S}}}_{2}\left({\varvec{t}}\right)/{{\varvec{S}}}_{2}({\varvec{a}})\right\}{\varvec{d}}{\varvec{t}}$$

where ***h***_***1***_**(*t*)** is the baseline age–specific hazard of developing breast cancer and ${{\varvec{S}}}_{2}\left({\varvec{t}}\right)={\varvec{e}}{\varvec{x}}{\varvec{p}}\left\{-{\int }_{0}^{1}{{\varvec{h}}}_{2}\left({\varvec{u}}\right){\varvec{d}}{\varvec{u}}\right\}$ is the probability of surviving competing risks up to age t [15].

The baseline age–specific hazard rates were obtained from the average (“composite”) age–specific breast cancer rates h*_1_(***t***) using ***h***_**1**_(***t***) = h*_1_(***t***)***F***(***t***), where ***F***(***t***) is 1 minus the attributable risk fraction for age t [16].

Statistical Package for the Social Science (SPSS) version 25 and Statistical Analysis Software (SAS) version 16 were used in calculations and significance testing.

Using the Gail Model as a golden standard, a woman with a probability of less than 1.66% of developing breast cancer in 5 years is considered to be at low risk. Conversely, a woman with a probability of more than 1.66% is classified as high risk and is recommended to undergo intensive screening by annual mammography and clinical breast examination every 6 to 12 months [15].

Regarding lifetime risk, a woman with a probability of less than 15% of developing breast cancer is considered to be at low risk, a woman with a probability of 15–30% is considered to be at moderate risk, and a woman with a probability of more than 30% is considered to be at high risk. Lifetime risk is defined as the risk of developing breast cancer up to 90 years of age [15].

## Results

The ages of the participants ranged from 35 to 84 years old, with a mean of 46.4 and a standard deviation of 9.5. The characteristics of the study participants are further clarified in Table 1.

**Table 1:** Characteristics of study participants

	Frequency	Percentage
**1. Are your parents blood relatives?**		
Yes	473	43.3
No	614	56.2
Unknown	6	0.5
**2. Marital status**		
Yes	1006	92
No	87	8
**3. Educational level**		
Illiterate	211	19.3
Primary	252	23.1
Secondary	293	26.8
University	337	30.8
**4. Number of children**		
No children	131	12
1–2 children	162	14.8
3–5 children	476	43.5
More than 5	324	29.6
**5. Age at first live birth**		
No birth	130	11.9
Less than 20 years	346	31.7
20–24 years	351	32.1
25–29 years	195	17.8
30–39 years	69	6.3
More than 40 years	2	0.2
**6. Breast feeding**		
Yes	846	77.4
No	247	22.6
**7. Use of contraceptives**		
Yes	544	49.8
No	549	50.2
**8. Age of menarche (first menstrual cycle)**		
7–11 years	113	10.3
12–13 years	671	61.4
More than 13 years	309	28.3
**9. Premenopausal of postmenopausal**		
Premenopausal	741	67.8
Postmenopausal	352	32.2
**10. Race**		
Arabic	881	80.6
Kurdish	137	12.5
Turkmen	75	6.9
**11. Monthly income to family**		
Low (Less than 250 IQD)	224	20.5
Moderate (250–Million IQD)	615	56.3
High (More than Million IQD)	254	23.2
**12. Smoking (including shisha and vape)**		
Yes	57	5.2
No	1036	94.8
**13. Physical activity**		
Never	648	59.3
Once weekly	188	17.2
Twice weekly	116	10.6
More than twice weekly	141	12.9
**14. Number of biopsies**		
No Biopsy	1058	96.8
One Biopsy (Without Hyperplasia)	35	3.2
**15. Number of first-degree relatives with breast cancer**		
No one	907	83
One relative	120	11
More than one relative	66	6
**16. Occupation**		
Housewife	736	67.3
Others	357	32.7

Table 2 shows the distribution of the participants throughout the Iraqi governorates.

**Table 2:** The governorates of the participants

	Frequency	Percentage
Anbar	46	4.2
Babil	45	4.1
Baghdad	191	17.5
Basra	97	8.9
Dhi Qar	75	6.9
Diyala	47	4.3
Dohuk	44	4
Erbil	53	4.8
Karbala	33	3
Kirkuk	58	5.3
Maysan	52	4.8
Muthanna	40	3.7
Najaf	62	5.7
Nineveh	60	5.5
Qadisiyyah	53	4.8
Saladin	51	4.7
Sulaymaniyah	37	3.4
Wasit	49	4.5
Total	1093	100

The mean of the 5-year risk was found to be 1.3 with a standard deviation of 1.0, and the mean of the lifetime risk was 13.4 with a standard deviation of 6.8. Table 3 lists the frequencies of the risks among the participants.

**Table 3:** The 5–year risk frequencies

	Frequency	Percentage
Low	823	75.3
High	270	24.7
Total	1093	100
**The lifetime risk frequencies**		
Low	707	64.7
Moderate	331	30.3
High	55	5.0
Total	1093	100.0

Table 4 shows the 5-year risk and the lifetime risk assessment measured using the Gail Model for the characteristics of study participants.

**Table 4:** Risk assessment using the Gail model

	5-year risk		Lifetime risk		
	Low	High	Low	Moderate	High
**1. Are your parents blood relatives?**					
Yes	364 (44.2%)	109 (40.3%)	295 (41.7%)	151 (45.7%)	27 (49%)
No	455 (55.2%)	159 (59%)	409 (57.8%)	177 (53.4%)	28 (51%)
Unknown	4 (0.6%)	2 (0.7%)	3 (0.5%)	3 (0.9%)	(0%)
**2. Marital status**					
Yes	741 (90%)	265 (98%)	625 (88.4%)	327 (98.8%)	54 (98.2%)
No	82 (10%)	5 (2%)	82 (11.6%)	4 (1.2%)	(1.8%)
**3. Educational level**					
Illiterate	114 (13.8%)	97 (36%)	131 (18.5%)	73 (22%)	7 (12.8%)
Primary	191 (23.2%)	61 (22.6%)	127 (18%)	113 (34.1%)	12 (21.8)
Secondary	238 (29%)	55 (20.3%)	177 (25%)	98 (29.6%)	18 (32.7%)
University	280 (34%)	57 (21.1%)	272 (38.5%)	47 (14.3%)	(32.7%)
**4. Number of children**					
No children	129 (15.6%)	2 (0.7%)	130 (18.3%)	1 (0.3%)	0 (0%)
1–2 children	127 (15.4%)	35 (13%)	98 (13.8%)	55 (16.6%)	9 (16.3%)
3–5 children	375 (45.5%)	101 (37.4%)	303 (42.8%)	141 (42.5%)	32 (58.1%)
More than 5	192 (23.5%)	132 (48.9%)	176 (25.1%)	134 (40.6%)	(25.6%)
**5. Age at first live birth**					
No birth	128 (15.6%)	2 (0.7%)	129 (18.2%)	1 (0.3%)	0 (0%)
Less than 20 years	181 (22%)	165 (61%)	70 (10%)	255 (77%)	21 (38.3%)
20–24 years	296 (36%)	55 (20.3%)	283 (40%)	43 (13%)	25 (45.4%)
25–29 years	169 (20.5%)	26 (9.6%)	169 (24%)	18 (5.4%)	8 (14.5%)
30–39 years	47 (5.7%)	22 (8.4%)	54 (7.6%)	14 (4.3%)	1 (1.8%)
More than 40 years	2 (0.2%)	0 (0%)	2 (0.2%)	0 (0%)	(0%)
**6. Breast feeding**					
Yes	617 (75%)	229 (84.8%)	515 (72.9%)	289 (87.4%)	42 (76.4%)
No	206 (25%)	41 (15.2%)	192 (27.1%)	42 (12.6%)	(23.6%)
**7. Use of contraceptives**					
Yes	424 (52.6%)	120 (44.4%)	314 (44.4%)	201 (60.7%)	29 (52.8%)
No	399 (48.4%)	150 (55.6%)	393 (55.6%)	130 (39.3%)	(47.2%)
**8. Age of menarche (first menstrual cycle)**					
7–11 years	63 (7.7%)	50 (18.2%)	52 (7.3%)	47 (14.1%)	14 (25.6%)
12–13 years	496 (60.2%)	175 (64.8%)	417 (59%)	219 (66.1%)	35 (63.4%)
More than 13 years	264 (32.1%)	45 (17%)	238 (33.7%)	65 (19.8%)	(11%)
**9. Premenopausal of postmenopausal**					
Premenopausal	179 (21.7%)	173 (21%)	445 (63%)	256 (77.4%)	40 (72.8%)
Postmenopausal	644 (78.3%)	97 (79%)	262 (37%)	75 (22.6%)	(27.2%)
**10. Race**					
Arabic	665 (80.8%)	216 (80%)	564 (79.7%)	271 (82%)	46 (83.6%)
Kurdish	99 (12%)	38 (14%)	89 (12.5%)	42 (12.6%)	6 (11%)
Turkmen	59 (7.2%)	16 (6%)	54 (7.8%)	18 (5.4%)	(5.4%)
**11. Monthly income to family**					
Low (Less than 250 IQD)	164 (20%)	60 (22%)	130 (18.5%)	81 (24.5%)	13 (23.8%)
Moderate (250–Million IQD)	459 (55.7%)	156 (58%)	385 (54.4%)	198 (59.8%)	32 (58.1%)
High (More than Million IQD)	200 (24.3%)	54 (20%)	192 (27.1%)	52 (15.7%)	(18.1%)
**12. Smoking (including shisha and vape)**					
Yes	32 (4%)	25 (9%)	40 (5.7%)	12 (3.7%)	5 (9%)
No	791 (96%)	245 (91%)	667 (94.3%)	319 (96.3%)	(91%)
**13. Physical activity**					
Never	473 (57.4%)	175 (65%)	421 (59.5%)	201 (60.7%)	26 (47.2%)
Once weekly	146 (17.7%)	42 (16%)	120 (17%)	58 (17.5%)	10 (18.1%)
Twice weekly	85 (10.4%)	31 (11%)	80 (11.3%)	25 (7.5%)	11 (20.2%)
More than twice weekly	119 (14.5%)	22 (8%)	86 (12.2%)	47 (14.3%)	(14.5%)
**14. Number of biopsies**					
No biopsy	804 (97.7%)	254 (94%)	16 (2.3%)	13 (4%)	6 (11%)
One biopsy (Without hyperplasia)	19 (2.3%)	16 (6%)	691 (97.7%)	318 (96%)	(39%)
**15. Number of first-degree relatives with breast cancer**					
No one	745 (90.5%)	162 (60%)	670 (94.7%)	235 (71%)	2 (3.7%)
One relative	68 (8.25%)	52 (19.25%)	31 (4.3%)	88 (26.5)	1 (1.8%)
More than one relative	10 (1.25%)	56 (20.75%)	6 (1%)	8 (2.5%)	52 (94.5%)

Table 5 shows the general linear regression model analysis predictors for both the 5–year and lifetime risks of developing breast cancer in Iraqi women between 35 and 85 years of age. The identified predictors for breast cancer in women were listed as age, age at menarche, age of first birth, number of first-degree relatives with breast cancer, race, number of biopsies, age of menopause, contraceptives usage, monthly income, smoking, and physical activity. Variables with (*P* < 0.001) were considered significant and strong predictors for breast cancer.

**Table 5:** Linear regression results for the 5-year and lifetime risks

	R-value	R^2^-value (%)	Standard error	*P* value
**5-year risk**				
Age	0.615	37.8	0.8156	< 0.001
Age of menarche	0.23	5.3	1.0065	< 0.001
Age at first birth	0.027	0.1	1.03387	0.369
Number of biopsies	0.124	1.5	1.02626	< 0.001
Race	0.013	0	1.03417	0.676
First degree relatives	0.546	29.8	0.8666	< 0.001
Menopause	0.459	21.1	0.9189	< 0.001
Using of contraceptives	0.044	0.2	1.033253	0.145
Monthly income	0.025	0.1	1.03393	0.406
Smoking	0.158	2.5	1.021231	0
Physical activity	0.055	0.3	1.032702	0.07
**Lifetime risk**				
Age	0.336	11.3	6.4536	0
Age of menarche	0.202	4.1	6.7098	0.003
Age at first birth	0.177	3.1	6.7427	0.01
Number of biopsies	0.002	0	6.851421	0.978
Race	0.047	0.2	6.84376	0.497
First degree relatives	0.753	56.8	4.50556	0
Menopause	0.280	7.8	6.57707	0
Using of contraceptives	0.065	0.4	6.83704	0.353
Monthly income	0.081	0.7	6.828912	0.245
Smoking	0.079	0.6	6.829924	0.256
Physical activity	0.067	0.4	6.836088	0.337

Globally reported Gail’s breast cancer risk assessments are classified in Table 6. The Gail Model overestimates risk in most of the studies outside the United States, due to the fact that the risk factors and incidence rates of breast cancer vary greatly across different races and countries.

**Table 6:** Gail's breast cancer risk in various countries

Country	Year	Sample size	Age	5–year risk	Lifetime risk
USA [13]	1989	4496	> 50	1.02	11.21
USA [17]	2001	319	≥ 35	1.67	–
USA [18]	2004	254	> 40	1.5	8.4
USA [19]	2005	8388	> 18	0.8	8
USA [20]	2006	99	≥ 35	4.13	23.5
USA [21]	2009	883	> 40	0.88	–
USA [22]	2016	124	> 50	1.67	–
Iraq (Baghdad) [23]	2016	250	≥ 35	0.95	11.3
Iraq (This Study)	2019	1093	≥ 35	1.33	13.36
Iran [24]	2008	2000	≥ 35	0.92	9.14
Iran [25]	2012	314	≥ 35	0.8	9
Iran [26]	2016	560	≥ 35	0.6	8.9
Iran [27]	2016	3847	≥ 35	1.61	11.71
Turkey [28]	2010	650	≥ 35	1.67	7.7
Turkey [29]	2011	415	> 20	1.7	15
Turkey [15]	2015	231	≥ 35	0.88	9.37
United Kingdom [15]	2013	355	> 46	1.5	9
Bulgaria [31]	2009	315	≥ 35	1.51	–
India [32]	2013	200	≥ 35	–	7.8
Korea [33]	2013	3789	< 50	0.44	2.24
Czech Republic [34]	2006	4598	≥ 35	1.37	8.02
Qatar [24]	2016	1488	≥ 35	1.12	10.57
Saudi Arabia [35]	2017	180	≥ 35	0.87	9.6
Bahrain [36]	2013	300	≥ 35	0.7	9.3

**Fig. 1:**
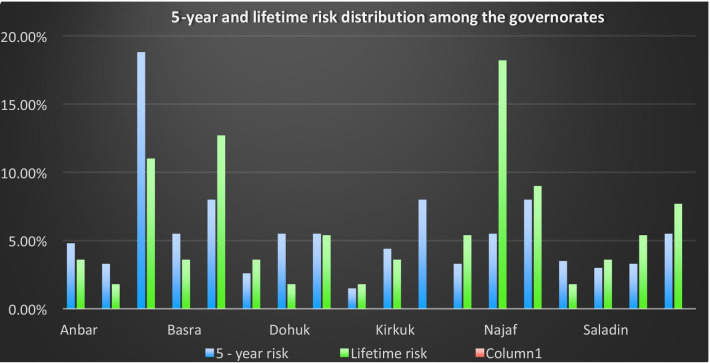
5-year and lifetime risk distribution among the governorates

Figure 1 shows the distribution of both risks on the governorates. It shows that Baghdad presents the highest 5-year risk, followed by Dhi Qar, Maysan, and Nineveh, in that order. Najaf presents the highest lifetime risk, followed by Dhi Qar, Baghdad, and Nineveh successively.

## Discussion

We found that the 5-year risk and lifetime risk of developing breast cancer for women in Iraq is 1.3 and 13.4, respectively. These figures are higher than those of a previous study performed in 2016 in Baghdad, which found a 5-year risk and lifetime risk of 0.95 and 11.3, respectively [23]. (For Baghdad only, we found 5-year and lifetime risk of 1.3 and 11.2, respectively, indicating that the 5-year risk increased but the lifetime risk stayed the same for the city).

Baghdad, Dhi Qar, Maysan, and Nineveh have the highest 5-year risk, while Najaf, Dhi Qar, Baghdad, and Nineveh have the highest lifetime risk.

Dhi Qar has a very high risk in both cases, so screening programs are especially important in this governorate; however, preventative measures should also be implemented in all.

According to the linear regression model, we found that age, family history, and menopause are the most important predictors for 5-year risk (with smoking playing a role as well), while family history is the most important predictor for lifetime risk.

Comparing data from studies performed in other countries (Table 6), Iraq has the highest breast cancer risk.

Prevention programs are therefore essential to eliminate high risk among Iraqi women, especially when one considers that breast cancer rates are increasing greatly throughout Iraq.

According to the Gail Model, older women have a greater risk of breast cancer—as age increases, the risk of BC increases. We found this to be true in our results, as risk was highest among the older age groups. This trend is similar to that of nearby countries, as shown in Table 6. We found that Iraq has the greatest risk of breast cancer among all countries, which may be due to the sociopolitical circumstances of the country (chemical warfare, bombings, etc.). This is likely why we see higher BC risk in the southern governorates such as Basra and Thi Qar, since these regions have historically been more prone to wars than the others (particularly in 1991 and 2003, when the wars were at their peak).

Women who take contraceptive pills should be aware of the fact that they are predisposed to breast cancer, according to the Gail Model and based on the results of our study. In addition, our study corrobated the association from previous studies that found women who do not breast feed their children are at a higher risk for BC.

We found women with a family history of breast cancer are at increased risk of developing breast cancer compared to women with no family history of breast cancer and recommend these women perform regular checks on themselves as a preventative measure. Moreover, there was no statistical difference in the effect of age on the demographic and clinical profiles of breast cancer among premenopausal versus postmenopausal Iraqi patients after controlling for marital status, level of education, and number of parities.

In Iraq, a significant proportion of breast cancer patients have a locally advanced disease at the time of diagnosis. To reinforce our national early detection program, it is essential to encourage public awareness through educational campaigns.

Screening programs and educational campaigns that teach Iraqi women to check their breasts regularly are crucial to limit this type of cancer. Considering that Iraq has a poor healthcare system and that many patients are deprived of high-quality care, it is imperative that preventative measures be discussed in order to reduce the incidence of breast cancer in the population [30].

## Limitations

It is worth mentioning the limitations that were observed while conducting this study. Chiefly, the Gail Model is calibrated to the United States’ population of women, and due to many variables, risk calculations might not be consistent for Iraqi women. Moreover, problems were encountered with sample randomization, since some women refused to participate, and some others had already been diagnosed with breast cancer.

## Conclusion

Breast cancer is a wide-spreading problem in the world and particularly in Iraq.

The Gail Model estimates the risk of developing breast cancer in any population, depending on its variables. The 5–year risk of BC among Iraqi women in 2019 was found to be distributed chiefly between Baghdad, Dhi Qar, Nineveh, and Maysan, with the greatest lifetime risk in Najaf and Dhi Qar. Screening programs are considered essential and heavily recommended in the process of breast cancer control and prevention. Prevention programs need to be implemented and awareness campaigns organized in order to highlight the importance of early detection and treatment to improve survival.

## Data Availability

The dataset can be obtained from the corresponding author upon reasonable request.

## Abbreviations

BC: Breast cancer
BCRAT: Breast Cancer Risk Assessment Tool
BRCA: Breast cancer gene
